# Monitoring the Persistence of *Pseudomonas sivasensis* Strain CF10PS3 in Cereal Fields

**DOI:** 10.1002/mbo3.70005

**Published:** 2024-11-18

**Authors:** Mathieu Delitte, Benjamin Dubois, Jacques Mahillon, Frédéric Debode, Claude Bragard

**Affiliations:** ^1^ Earth and Life Institute—Applied Microbiology, Plant Health UCLouvain Louvain‐la‐Neuve Belgium; ^2^ Earth and Life Institute—Applied Microbiology, Food and Environmental Microbiology UCLouvain Louvain‐la‐Neuve Belgium; ^3^ Bioengineering Unit, Life Sciences Department Walloon Agricultural Research Center Gembloux Belgium

**Keywords:** biocontrol agents (BCAs), environmental impact, microbial persistence, phyllosphere, plant‐microbe interactions, *Pseudomonas sivasensis*, qPCR monitoring, sustainable agriculture, wheat microbiome

## Abstract

The persistence and efficacy of biocontrol agents in agricultural fields are crucial for sustainable crop production. In this study, we investigated the persistence of the introduced bacterial strain *Pseudomonas sivasensis* CF10PS3 in the wheat phyllosphere using a novel qPCR probe protocol. The CF10PS3 strain, known for its in vitro biocontrol properties against wheat pathogens, was applied through foliar spray, and its persistence was monitored over 7 weeks. Our qPCR assays, designed to specifically detect CF10PS3, distinguished it from naturally occurring *P. sivasensis* strains, providing precise insights into its dynamics in the field. The experimental results indicated that CF10PS3 was already present on the wheat leaves before its application, suggesting its natural adaptation to the foliar environment. Following initial application, a significant increase in CF10PS3 was observed, though subsequent environmental factors such as rain and wind might have caused notable fluctuations in its population. Despite these variations, the introduced strain showed considerable persistence, with population levels significantly higher than those in untreated plots by the end of the study period. This research underscores the importance of understanding bacteria dynamics in the field, highlighting the influence of environmental conditions on their efficacy. The use of specific qPCR probes proved effective in monitoring introduced strains, offering valuable insights for optimizing biocontrol agent application strategies. Our findings contribute to the development of robust biocontrol methods, promoting sustainable agricultural practices and enhancing crop protection.

## Graphical abstract

This study developed qPCR probes that specifically distinguish *Pseudomonas sivasensis* CF10PS3 from other strains within the species. These probes enabled precise monitoring of CF10PS3 persistence in the wheat phyllosphere under field conditions, providing insights into microbial population dynamics.

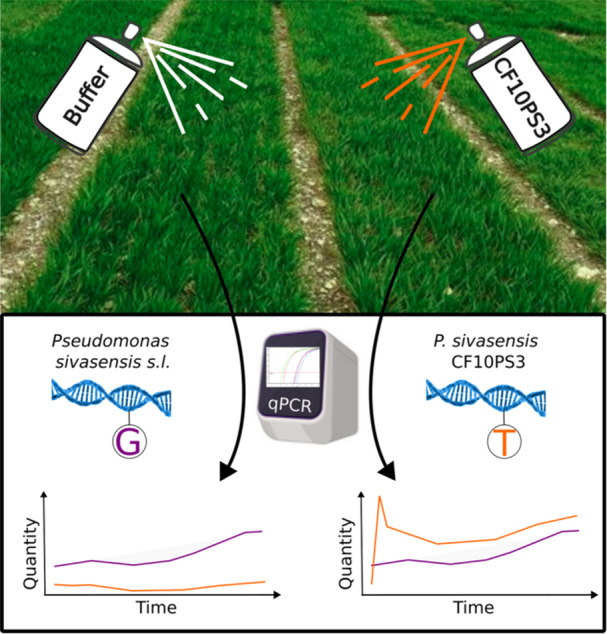

## 1 Introduction

The use of beneficial microorganisms in agriculture has recently gained significant attention as an eco‐friendly and sustainable approach. They allow for enhancing crop productivity and reduce the environmental impact of chemical treatments. *Pseudomonas* spp. strains have emerged as pivotal beneficial microorganisms due to their diverse array of plant growth‐promoting and biocontrol capabilities (Mehmood et al. 2023). They have exhibited remarkable potential for increasing crop yields, improving soil health, and protecting plants against phytopathogens (Martínez et al. 2019; Ghadamgahi et al. 2022; Ridene et al. 2023). Despite such promising abilities, to date, no biocontrol agent has consistently demonstrated effective control of wheat foliar diseases, particularly *Zymoseptoria tritici*, the causative agent of Septoria tritici Blotch (STB).

In the phyllosphere, encompassing the above‐ground parts of plants, such as leaves and stems, the survival and colonization of *Pseudomonas* spp. strains are subject to a complex interplay of environmental factors (Chaudhry et al. 2021). High humidity levels often create a more favorable environment by reducing desiccation stress on the leaf surfaces, thus promoting microbial growth (Aung, Jiang, and He 2018). However, the relationship between humidity and microbial colonization is also influenced by additional factors. Temperature fluctuations, for example, modulate the metabolic activity and growth rates of *Pseudomonas* spp. (Kahl et al. 2022; Bisht, Luecke, and Wakeman 2023). Rainfall events introduce another dimension, potentially washing away or diluting microbial populations, but also providing moisture essential for survival (Pietrarelli, Balestra, and Varvaro 2006). Moreover, the presence of other microorganisms on leaf surfaces, forming the phytobiome, and the specific characteristics of the plant host further influence the dynamics of microbial communities (Leach et al. 2017; Sivakumar et al. 2020; Zhang et al. 2021; Bashir et al. 2022). Consequently, the interaction between *Pseudomonas* spp. and the phyllosphere is a multifaceted process, where humidity, temperature, rain, and a myriad of ecological factors collectively shape the outcomes of microbial colonization and its potential biocontrol activities against plant pathogens. Understanding this intricate balance is pivotal for harnessing the full potential of *Pseudomonas*‐based strategies in sustainable agriculture.

The successful use of *Pseudomonas* spp. in agricultural field trials necessitates the development of robust tools for their detection, quantification, and monitoring. Understanding the presence and persistence of these strains in the field is essential for optimizing their application strategies, but also for assessing their long‐term impacts on both crop health and the broader ecosystem. Traditional culture‐based methods and generic molecular techniques have been valuable in elucidating the presence of *Pseudomonas* populations in soil and plant‐associated niches (Elad and Kirshner 1993; Paul et al. 2017; Pujol et al. 2006). However, they often lack the specificity required to discriminate between closely related *Pseudomonas* strains, limiting their use in strain‐specific monitoring.

To address this limitation, we present in this study the design and validation of a strain‐specific probe tailored for the precise detection of a *Pseudomonas* strain used in a recent agricultural field trial. The development of such a probe represents a critical step toward elucidating the fate and dynamics of this specific strain in the complex environment of cereal fields.

Here, we describe the rational design of the strain‐specific probe, its molecular validation, and its application up to the field trial stage. We also discuss the broader implications of our findings for the use of Pseudomonads in sustainable agriculture and the advantages of strain‐specific monitoring approaches.

The ability to selectively detect and monitor specific *Pseudomonas* sp. in agricultural field trials holds promise for enhancing our understanding of their behavior in natural ecosystems and optimizing their application for sustainable crop production. Furthermore, it opens up new avenues for tailoring biocontrol and plant growth‐promoting strategies based on an in‐depth understanding of strain‐specific interactions with the crop.

## 2 Materials and Methods

### 2.1 Bacterial Strain and Formulation

*Pseudomonas* isolate CF10PS3 was obtained from a flag leaf in a spelt field (*Triticum spelta*) located in Walhain, Belgium. The field was managed under organic agriculture, without tillage. The isolate was identified as *Pseudomonas sivasensis* strain CF10PS3. Briefly, identification was performed through multi‐locus sequence analysis targeting 16S rRNA, ileS, gapA, glnS, gltA, gyrB, nuoD, recA, rpoB, and rpoD genes and later confirmed via whole‐genome sequencing performed with both Illumina and MinION technologies. In vitro assessments revealed the strain's ability to produce siderophores, proteases, and cellulase, as well as its ability to solubilize phosphate and exhibit swimming and swarming behaviors. It has also shown a great ability to colonize wheat leaves both through direct spray applications and through translocation from seed coating. Both in vitro and in planta studies demonstrated CF10PS3's efficacy against *Z. tritici*, the causal agent of STB in wheat. Strain CF10PS3 possesses the genetic determinants for the production of several biocontrol‐related metabolites. These include viscosin, fragin, pseudopyronine, the aryl polyene APE‐Vf, koreenceine, and siderophore. The strain is available via the LMG collection in Belgium, under collection number LMG S‐33705. It can also be obtained through a Material Transfer Agreement (MTA) from the Plant Health Laboratory at the Earth and Life Institute—Applied Microbiology, UCLouvain.

Before each field application, CF10PS3 was grown on lysogeny broth (LB) agar plates at 28°C for 24 h (10 g/L tryptone, 5 g/L yeast extract, 5 g/L NaCl). A single colony was selected and inoculated into 20 mL of LB. Following 24 h of growth at 30°C, 180 rpm, the bacterial suspension was used to inoculate 2 L of LB, incubated at 30°C, 180 rpm for 24 h. Following incubation, the bacterial suspension was centrifuged and the bacterial pellets were resuspended in the buffer to reach a final concentration of 10^7^ CFU/mL. The buffer consisted of 10 mM MgCl_2_ and 0.1% Tween 20. All liquids were sterilized by autoclaving at 121°C, 3 bars for 1 h. Sterile buffer was used as a negative control in mock treatment.

### 2.2 Plant Treatment and Sampling

Winter wheat cultivar Chevignon was sown in an agricultural field in Perwez, Belgium, on October 15, 2022. The sowing followed a potato crop rotation. Trial plots, each measuring 10 × 2 m, were randomly assigned to four replicates per treatment modality. Bacteria suspended in buffer, as well as sterile buffer alone (which will be referred to as the mock‐treatment), were applied to the corresponding plots on April 27, 2023 (BBCH32) to protect the third leaf, on May 11, 2023 (BBCH37) to protect the second leaf, and on May 17, 2023 (BBCH39) to protect the flag leaf. The BBCH scale provides a standardized method for describing phenological development (Meier et al. 2009). Leaf sampling was conducted by cutting the base of seven leaves within each plot. The harvested leaves were immediately placed in sterile Falcon tubes and stored on ice until they could be transferred to a laboratory for weighing and freezing at −20°C. Leaf samplings were performed following this schedule: for the second leaf, sampling occurred before and 1 h after treatment, as well as at 1, 4, and 6 days posttreatment. On the sixth day after the second leaf treatment, additional bacterial application was made to protect the first leaf. Consequently, both the first (F1) and second (F2) leaves were sampled at each designated sampling time: before and 1 h after F1 treatment, 2 days after F1 treatment, 5, 7, 14, 22, 29, 36, 43, and 50 days after the F1 treatment. This comprehensive sampling approach allowed detailed monitoring of *P. sivasensis* strain CF10PS3 dynamics in the wheat field.

### 2.3 Harvest of Epiphytic Microbial Communities and DNA Extraction

To recover the diverse epiphytic microbial communities present on the leaves, we adopted the leaf harvest protocol as follows: leaves were placed in sterile 250 mL wide‐neck Erlenmeyer flasks, each containing 50 mL of a sterile peptone‐phosphate buffer (peptone 10 g/L, NaCl 5 g/L, KH₂PO₄ 7.5 g/L, and KH₂PO₄·3H₂O 9.5 g/L). Following an incubation period of 30 min at room temperature, the Erlenmeyer flasks were subjected to agitation at 150 rpm for 75 min. This was followed by a 45‐min resting period at room temperature. The liquid from each flask was then transferred back into the initial Falcon tubes and centrifuged for 20 min at 6000 rpm. Subsequently, the supernatant was discarded, while the pelleted microorganisms were stored at −20°C for subsequent analysis.

DNA extraction was performed with the DNeasy PowerSoil Pro Kit (Qiagen), following the manufacturer's protocol with one modification: the initial lysis buffer was used to resuspend the pellet. The resuspended pellet was then placed into the Power‐Beads tube, and the remainder of the protocol proceeded without further changes. Extracted DNA was quantified using the Qubit4 system (Thermo Fisher Scientific).

### 2.4 Probes and Primer Design

A single‐nucleotide polymorphism (SNP) was identified in the alignment of the 319 rrn sequences (16S‐ITS‐23S operon) obtained from NCBI complete genomes of wheat‐associated *Pseudomonas*, and the complete sequenced genome of isolate CF10PS3 (Table A1). *P. chlororaphis* was also considered as it is one of the two commercialized *Pseudomonas* strains in Belgium. The other one is of an undetermined species (strain DSMZ 13134); it was not considered in this study. To specifically detect the presence of *P. sivasensis* strain CF10PS3 among bacterial communities in leaf‐washing samples, a set of two primers was designed to amplify the region encompassing the targeted SNP (either a “T” in isolate CF10PS3 or a “G” in all the other sequences) in the rrn operon. Complete alignment of the 319 sequences can be found in the Zenodo repository: https://doi.org/10.5281/zenodo.12633665.

The TaqMan probe sequence was custom‐designed by adjusting the size and number/position of locked nucleic acid (LNA) bases to enhance its affinity for the specific target sequence (https://eu.idtdna.com/calc/analyzer) while minimizing stability toward nontarget DNA sequences, allowing for effective mismatch discrimination (You et al. 2006). Primers were selected using the Primer3 web tool (http://bioinfo.ut.ee/primer3-0.4.0/) to amplify a region of 100–200 bp surrounding the probe sequence, ensuring optimal compatibility with the designed probes (Table 1).

**Table 1:** Primers and probes characteristics.

Primers and probes characteristics	Sequence (5′ > 3′)	*T*_m_ (°C)	Amplicon length (bp)
Ps_sivasensis_F	CGTTTGGCTCCACCACTACT	60	145
Ps_sivasensis_R	ACGTTCAGTCTATCTTTCTATCACA	58	
Ps_sivasensis_T	FAM‐CATTGT**T**ATGATGGTGAA‐BHQ1	61.1	
Ps_sivasensis_G	HEX‐CATTGTGATGATGGTGAA‐BHQ1	61.1	

*Note:* Underscored bases are locked nucleic acid (LNA) and bases in bold are the SNPs specific for each probe. Melting temperatures (*T*_m_) are indicated in Celsius degrees. FAM and HEX are the fluorophores used with the BHQ‐1 quencher.

The compatibility of all designed primers to work in a single reaction was evaluated using the multiple primer analyzer web tool (www.thermofisher.com). The specificity of the probe and primer pair was verified on GenBank using primer‐Blast and Blastn algorithms (Ye et al. 2012). The combination of T‐probe and primers only hits the targeted *P. sivasensis* CF10PS3. The G‐probe and primers combination allowed for the detection of all targeted *P. sivasensis* strains (reference strains 2RO45, BsEB1, P7, CCUG57209, W6, and strains BE10PS1, BE10PS3).

### 2.5 Cloning of the Target DNA and Determination of Plasmid Copy Number

To produce amplicons representative of the target sequences, the primer pair was used in a standard PCR reaction with Q5 High Fidelity DNA Polymerase (NEB) and DNA extracted from *P. sivasensis* strain CF10PS3 (for T‐SNP) and strain DS3PS5 (for G‐SNP). The resulting PCR products were visualized on an agarose gel to ensure specificity and subsequently purified using a SmartPure PCR kit (Eurogentec). DNA purity and concentration were assessed with a NanoDrop spectrophotometer (Thermo Fisher Scientific) and a Quantus fluorometer (Promega). To standardize PCR reactions, DNA fragments with either T or G SNP were ligated into pK18mobsacB plasmid following the Gibson Assembly Cloning Kit instructions (NEB) and introduced in competent *E. coli* using the heat‐shock transformation protocol. Briefly, 10 μL of Gibson Assembly products were added to 200 μL of competent cells. The mixture was kept on ice for 15 min before going to a 42°C bath for 42 s and getting back on ice for 2 min. The mixture was supplemented with 950 μL of LB medium and incubated at 37°C, 150 rpm, for 1 h. Bacteria were concentrated by centrifugation (5000*g*, 10 min) and spread on LBA plates containing kanamycin, X‐gal, and IPTG. Plasmid DNA isolated from bacterial cultures was extracted with Qiaprep Spin Miniprep Kit (Qiagen) and adjusted to 10^8^ copies with Thermo Fisher DNA Copy Number and Dilution Calculator with a molar mass per base pair set at 650 Da. The limit of detection, robustness, and efficiency were determined on diluted plasmid DNA.

### 2.6 Limit of Detection

The absolute limit of detection (LOD) for our PCR assay, which includes primers, both probes and the amplification program, was determined using serial dilutions of plasmid DNA. For these dilutions, we aimed for target copy numbers of 50, 20, 10, 5, 2, 1, and 0.1. We performed six PCR reactions for every dilution level. The LOD_6_, defined as the lowest number of copies at which all six PCR tests yield positive results, was identified under the condition that the highest dilution, supposedly containing 0.1 copy per reaction, resulted in no more than one positive PCR out of six attempts (Association Française de Normalisation AFNOR 2003). We subjected the copy number at the LOD_6_ to 60 additional tests within the same run to determine the LOD_95_. The LOD_95_ criterion is met when at least 95% of these 60 tests produce positive results. The maximum acceptable copy number for both LOD_6_ and LOD_95_ were set at 20 copies.

### 2.7 Dynamic Linear Range and Efficiency

The dynamic range of our PCR assay was evaluated over eight orders of magnitude ranging from 10^8^ to 10^1^ copies. For each dilution, we conducted the assay in six replicates. We analyzed linearity by plotting the cycle threshold (Ct) values against the logarithmic scale of target concentrations (Stolovitzky and Cecchi 1996). Dilutions from 10^8^ to 10^1^ copies in six replicates were also used in two plates, as well as in duplicates in eight other plates, to calculate PCR efficiency. To be considered acceptable, efficiency has to be between 90% and 110% (Broeders et al. 2014).

### 2.8 Transferability and Robustness

To assess the transferability of the method, LOD_6_, LOD_95_, linear range, and efficiency were tested following the same protocols in another independent laboratory at the Walloon Agricultural Research Centre (CRA‐W), located in Gembloux, Belgium. The robustness of our method was evaluated by implementing variations to the standard experimental procedures (Commission 2010). The parameters we adjusted included the primer concentrations (either standard or reduced by 30%), probe concentrations (standard or reduced by 30%), and the volume of the real‐time PCR Master Mix (standard or altered by ±1 μL), resulting in a total reaction volume of 20 ± 1 μL. We conducted six replicates containing 20 copies of each plasmid target, under the modified conditions detailed in Table 2. The key criterion for acceptance was that all alterations to the standard protocol should consistently yield a positive result at a target level of 20 copies per reaction, as per the guidelines defined by Broeders et al. (2014).

**Table 2:** Experimental conditions tested to evaluate robustness of the method.

PCR parameter	Standard condition	Tested variation
PCR machine	CFX96 thermocycler (Bio‐Rad)	—
PCR reagent kit	Takyon No Rox Probe 2X MasterMix dTTP (Eurogentec)	—
Primer concentrations	Standard	Minus 30%
Probe concentrations	Standard	Minus 30%
PCR volume	Standard (18 µL mix + 2 µL DNA)	19 µL mix + 2 µL DNA/17 µL mix + 2 µL DNA

*Note:* Variations to the standard protocol tested in the robustness evaluation. The standard protocol is in the second column. Primers (−30%) and probes (−30%) concentration limitations were tested, as well as the reaction volume (±1 μL of Master Mix)

### 2.9 Specificity

The specificity of the method was checked on 34 organisms from different taxonomic bacterial genera, including 20 *Pseudomonas* spp. isolates. The possibility of cross‐reaction with human DNA was considered, and host wheat DNA was also checked. Briefly, human DNA was extracted from saliva and wheat DNA from a young leaf obtained from disinfected seed grown in sterile conditions and surface‐disinfected before extraction. Both DNA samples were extracted using the DNeasy PowerSoil Pro Kit (Qiagen), following the manufacturer's recommendations. Each DNA extract was tested at least in duplicate. Two nanograms of DNA were used in the PCRs. The tested organisms are outlined in Table 3. Except for *P. aeruginosa* and *P. paraeruginosa*, which were obtained from humans, all other organisms were isolated from agricultural environments, primarily associated with wheat, and all DNA was also extracted with the PowerSoil DNA Extraction Kit (MO BIO Laboratories, USA), following the manufacturer's recommendations.

**Table 3:** List of organisms used to evaluate qPCR specificity.

Genus	Species	Strain	Origin	Collection number
*Bacillus*	*subtilis*	BF12B1	Wheat leaf	UPB 1356
*Brevundimonas*	*alba*	18360	Soil	LMG 18360
*Burkholderia*	*anthina*	22950	Maize rhizosphere	LMG 22950
*Enterobacter*	*kobei*	BC B1	Field soil	UPB 1352
*Flavobacterium*	*johnsoniae*	LMG1341	Soil	LMG1341
*Glutamicibacter*	*creatinolyticus*	BC F7	Field soil	UPB 1350
*Homo*	*sapiens*			
*Methylobacterium*	*bullatum*	24788	Moss phyllosphere	LMG 24788
*Microbacterium*	*oxydans*	E109	Field soil	UPB 460
*Pantoea*	*agglomerans*	Pa19	Wheat head	UPB 1357
*Pedobacter*	*foliorum*	31463	Field soil	LMG 31463
*Pseudomonas*	*aeruginosa*	Pa01	Wound	LMG 1242
*Pseudomonas*	*cichorii*	P14	Field soil	UPB 461
*Pseudomonas*	*cyclaminis*	AR12PS3	Wheat root	UPB 1358
*Pseudomonas*	*fuscovaginae*	CB51	Wheat	UPB 526
*Pseudomonas*	*lurida*	BF10PS1	Wheat leaf	UPB 1359
*Pseudomonas*	*lurida*	BF8PS1	Wheat leaf	UPB 1355
*Pseudomonas*	*marginalis*	DR4PS3	Wheat root	UPB 1360
*Pseudomonas*	*arvensis* sp. nov.	CR7PS1	Wheat root	UPB 1361
*Pseudomonas*	*arvensis* sp. nov.	DR1PS3	Wheat root	UPB 1362
*Pseudomonas*	*arvensis* sp. nov.	DS3PS4	Field soil	UPB 1363
*Pseudomonas*	*arvensis* sp. nov.	DS3PS5	Field soil	UPB 1364
*Pseudomonas*	*orientalis*	CF11PS1	Wheat leaf	UPB 1365
*Pseudomonas*	*paraeruginosa*	8029	Infected ear	LMG8029
*Pseudomonas*	*protegens*	44RP8	Field soil	UPB 1366
*Pseudomonas*	*sivasensis*	BE10PS1	Wheat head	UPB 1367
*Pseudomonas*	*sivasensis*	BE10PS3	Wheat head	UPB 1368
*Pseudomonas*	*sivasensis*	CF10PS3	Wheat leaf	UPB 1354
*Sphingobacterium*	*thalpophilum*	BC B3	Field soil	UPB 1353
*Sphingomonas*	*albertensis*	32139	Wheat leaf	LMG 32139
*Staphylococcus*	*equorum*	BC G2	Field soil	UPB 1351
*Triticum*	*aestivum*			
*Xanthomonas*	*translucens* pv. *undulosa*	CB4	Wheat	UPB 513

### 2.10 qPCR Assay for Bacteria Quantification

The mixture for qPCR assay was prepared with Takyon No ROX Probe 2X MasterMix dTTP (Eurogentec), 300 and 200 nM of each primer and probe, respectively, and 2 μL of DNA template in a total volume of 20 μL. The amplification reaction was performed on a CFX96 thermocycler (Bio‐Rad). The qPCR cycling protocol included an activation step at 95°C for 3 min, followed by 40 cycles of denaturation at 95°C for 10 s and annealing/extension at 60°C for 45 s, with fluorescence intensity measured at the end of each cycle. Each sample was run in two technical replicates. Eight 10‐fold serial dilutions of each plasmid (ranging from 10^8^ to 10^1^ DNA copies) were prepared and used as templates for each qPCR run. These dilutions were utilized to generate calibration curves, correlating known starting quantities with their corresponding Ct (cycle threshold) values. These calibration curves were instrumental in achieving absolute quantification of the copies from both the T and G probes in the samples by plotting the Ct values against the established calibration curve.

### 2.11 qPCR Assay for Wheat DNA Quantification

To assess the presence of plant DNA contaminants in DNA extracts, we conducted qPCR assays targeting the wheat ARF and DUF52 housekeeping genes. These genes were chosen due to their demonstrated stability in terms of expression within wheat genomes. Briefly, the qPCR reactions employing TaqMan probes were prepared using 10 μL of Takyon No ROX Probe 2x Mastermix dTTP Blue (Eurogentec), with a concentration of 300 nM for each primer and 100 nM for the TaqMan probe labeled with the FAM fluorophore and TAMRA quencher (Eurofins Genomics). Additionally, 2 μL of the extracted DNA and nuclease‐free water (Thermo Scientific) were added to reach a total reaction volume of 20 μL. The prepared samples were loaded into a Hard‐Shell 96‐well PCR skirted white plate and securely sealed using a Microseal'B’ PCR Plate Sealing Film (Bio‐Rad). Subsequent PCR amplifications were conducted on the C1000 TouchTM thermal cycler coupled with the CFX96TM Real‐Time detection system (Bio‐Rad). The thermal cycling program involved an initial denaturation step at 95°C for 3 min, followed by 40 cycles of denaturation at 95°C for 10 s and annealing/extension at 69°C for 1 min. Each sample was subjected to two technical replicates to ensure precision and reliability. For the quantification of relative wheat DNA content in each sample, we used the 2^∆Ct^ method, where ∆Ct represents the difference between the Ct values for the wheat (Ct_wheat) and the sample (Ct_sample). DNA extracted from thoroughly disinfected leaves served as a reference for “pure” wheat DNA content. This method allowed us to accurately estimate the proportion of wheat DNA in the samples.

### 2.12 Data Analysis

All data analyses were performed using R Studio (version 2022.07.1 Build 554). The initial steps were to set the limit of detection to the one calculated in previous steps and normalize DNA concentrations. To achieve this, the qPCR data for each wheat gene (ARF and DUF52) were processed separately. The mean Ct values of technical replicates were computed for each sample, as well as for the “pure” wheat DNA extract. ∆Ct values were calculated by subtracting the mean Ct value of wheat from each Ct value of the samples. The relative abundance of wheat DNA in the samples was then calculated using the 2^∆Ct^ method. The final relative abundance (Relab) of wheat was determined by averaging the relative abundance values obtained from each gene for each sample. DNA concentration (DNA cc) in each sample was subsequently normalized (nDNA) as follows: nDNA = (1 − Relab) × DNA cc.

Data obtained from qPCR assays targeting the FAM and HEX probes, which detect copies from CF10PS3 strain or the natural population, respectively, were utilized as “Starting Quantities,” divided by the mass of the sample to express data by a gram of tissue. All data beyond the LOD (see Section 2.6.) were discarded. The means of both values for technical replicates were calculated for each fluorophore and each sample, and normalization was carried out, taking into account the nDNA values calculated earlier. Additionally, the fluorophore copies for each treatment were averaged across the four field replicates, and their standard error values were calculated. Logarithms of these data were calculated to facilitate their representation in graphical form. The normality of the data was assessed by the Shapiro–Wilk normality test with *p* < 0.05. For statistical analysis, a mixed linear model was built for each foliar stage to take into account the dynamics of samplings. Hourly meteorological data used in the analysis were sourced from the CRA‐W Gembloux meteorological station, 10 km away from the field.

## 3 Results

### 3.1 Strain Characterization

*P. sivasensis* is found as a common bacterial species associated with small‐grain cereals (spelt, wheat). *P. sivasensis* strain CF10PS3 was isolated from a flag leaf in a spelt field in Walhain, Belgium, managed under organic agriculture. This strain was selected for its potential biocontrol‐related metabolites production, its colonization abilities on wheat leaves, and its potential control of STB. Interestingly, based on an alignment of 319 *Pseudomonas* spp. strains (Table A1), CF10PS3 can be distinguished from the other ones usually found in wheat fields, as it has a “T” SNP in the 16S‐ITS‐23S operon that allowed us to design a qPCR experiment to specifically detect it. Other sequences from *P. sivasensis* and closely related species (*P. arvensis* sp. nov. and *P. cyclaminis*) have a “G” SNP and are detected with the primers and G‐probe combination. All other sequences were theoretically verified in silico and were neither amplified by the primers nor detected by the probes, except for 11 sequences among the 319, belonging to *P. fluorescens* and *P. poae* species and representing a low‐level percentage of off‐target detection (< 3.5%; Table A1).

### 3.2 Method Performances

We assessed the performance of the designed experiment across various parameters to ensure accuracy and reliability. This includes evaluating the specificity, limit of detection, linearity, efficiency, and robustness, ensuring trustworthy results for the intended application.

The specificity of the method was checked on 34 organisms related to our case study. Human DNA was checked for cross‐contamination. Wheat DNA was verified for its lack of influence on the experiment. The probe labeled with the FAM fluorophore only gave positive results for P*. sivasensis* CF10PS3, confirming the “T” SNP is only present in that strain (Table 4, FAM column). The other probe, labeled with the HEX fluorophore and designed to detect the “G” SNP, effectively gave positive responses for all other *P. sivasensis* sensu lato, confirming its relevance in evaluating the natural population of that group (Table 4, HEX column).

**Table 4:** Specificity of the method.

Target	Results FAM	Results HEX
*P. sivasensis* CF10PS3	+(*m* = 15.74 +/0.17)	−
*P. sivasensis* BE10PS1	−	+(*m* = 17.92+/0.16)
*P. sivasensis* BE10PS3	−	+(*m* = 18.78+/0.48)
*P. cyclaminis* AR12PS3	−	+(*m* = 17.84+/0.15)
*P. arvensis* sp. nov. CR7PS1	−	+(*m* = 18.58+/0.03)
*P. arvensis* sp. nov. DR1PS3	−	+(*m* = 19.02+/0.11)
*P. arvensis* sp. nov. DS3PS4	−	+ (*m* = 19.64+/1.28)
*P. arvensis* sp. nov. DS3PS5	−	+ (*m* = 19.31+/0.05)
*Bacillus subtilis* BF12B1	−	−
*Brevundimonas alba* LMG 18360	−	−
*Burkholderia anthina* LMG 22950	−	−
*Enterobacter tabaci* BC B1	−	−
*Flavobacterium johnsoniae* LMG1341	−	−
*Glutamicibacter creatinolyticus* BC F7	−	−
*Methylobacterium bullatum* 24788	−	−
*Microbacterium oxydans* E109	−	−
*P. aeruginosae* Pa01	−	−
*P. cichorii* P14	−	−
*P. fuscovaginae* CB51	−	−
*P. lurida* BF10PS1	−	−
*P. lurida* BF8PS1	−	−
*P. marginalis* DR4PS3	−	−
*P. orientalis* CF11PS1	−	−
*P. paraeruginosae* 8029	−	−
*P. protegens* 44RP8	−	−
*P. syringae* pv. *aptata* 5143	−	−
*P. syringae* pv. *atrofaciens* P64	−	−
*P. viridiflora* 2352	−	−
*Pantoea agglomerans* 19	−	−
*Pedobacter foliorum* 31463	−	−
*Sphingobacterium thalpophilum* BC B3	−	−
*Sphingomonas albertensis* 32139	−	−
*Staphylococcus equorum* BC G2	−	−
*Xanthomonas translucens* pv. *undulosa* CB4	−	−
Human DNA	−	−
Wheat DNA	−	−

*Note:* += positive signal; − = no signal.

For positive samples, mean Cq values with their standard deviations are given in brackets.

This experimental setup confirmed in silico results where the combination of primers and probes allowed specific detection of either CF10PS3 or the other *P. sivasensis s.l.* The limit of detection was evaluated following two complementary approaches. The LOD_6_ of the method was estimated at 20 copies for each target, since this quantity was detected in all six replicates, and the highest dilution, supposedly containing 0.1 copy per reaction, resulted in no more than one positive PCR out of six attempts, as required in Broeders et al. (2014). For LOD_95_, at least 57 out of 60 attempts yielded positive results with both probes on target plasmid DNA.

**Figure 1:**
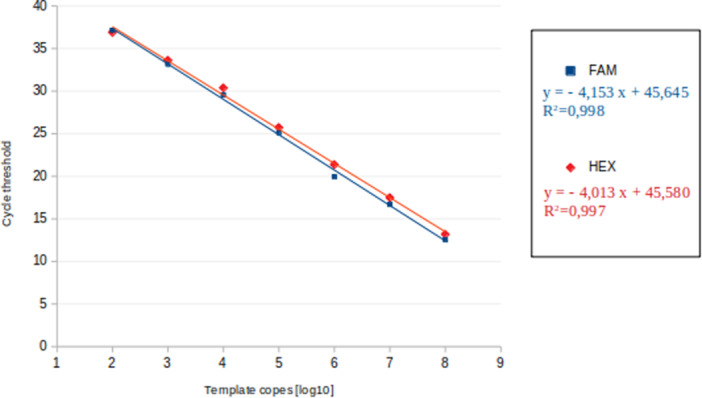
Linearity of the designed qPCR. This was analyzed with serial dilutions from 10^8^ to 10^2^ copies of both probes (labeled with FAM fluorophore in blue or HEX fluorophore in red). Points represent the mean values of six replicates. Lines represent linear fits of the data with their associated equations of calibration function and correlation coefficients.

Linearity was observed over a range from 10^2^ to 10^8^ copies per reaction (Figure 1). The coefficient of determination (*R*²) was calculated as > 0.99 for both probes. At 10 copies, only three of the six replicates were positive for each probe, since this number is under the LOD and therefore was not considered for linearity evaluation.

For each plate, efficiency ranged between 90% and 110%, meeting the criteria proposed by Broeders et al. (2014). The PCR method robustness was successfully evaluated on plasmid DNA. All tests with deviations from the standard protocol delivered positive results with 20 template copies, with only slight deviations from the expected Cq value (Table 5), aligning with the requirements described in Broeders et al. (2014).

**Table 5:** Robustness of the qPCR method.

	FAM fluorophore			HEX fluorophore		
Deviations from standard protocol	Expected Cq	Observed Cq	Coefficient of variation (%)	Expected Cq	Observed Cq	Coefficient of variation (%)
−30% primers	35.19	34.5	2.05	36.47	35.1	3.68
−30% probes	35.19	35.9	−2.11	36.47	36.5	−0.19
−1% Master‐Mix	35.19	35.4	−0.48	36.47	37.1	−1.68
+1% Master‐Mix	35.19	35.3	−0.29	36.47	37.3	−2.17

### 3.3 Persistence of Introduced *P. sivasensis* in Wheat Phyllosphere

The newly developed qPCR protocol was then used to investigate the persistence of *P. sivasensis* CF10PS3 in the wheat phyllosphere over key wheat growth stages following its application via foliar spray. A mock treatment consisting of the sterile buffer alone was also evaluated for comparison.

#### 3.3.1 Initial Situation

**Figure 2:**
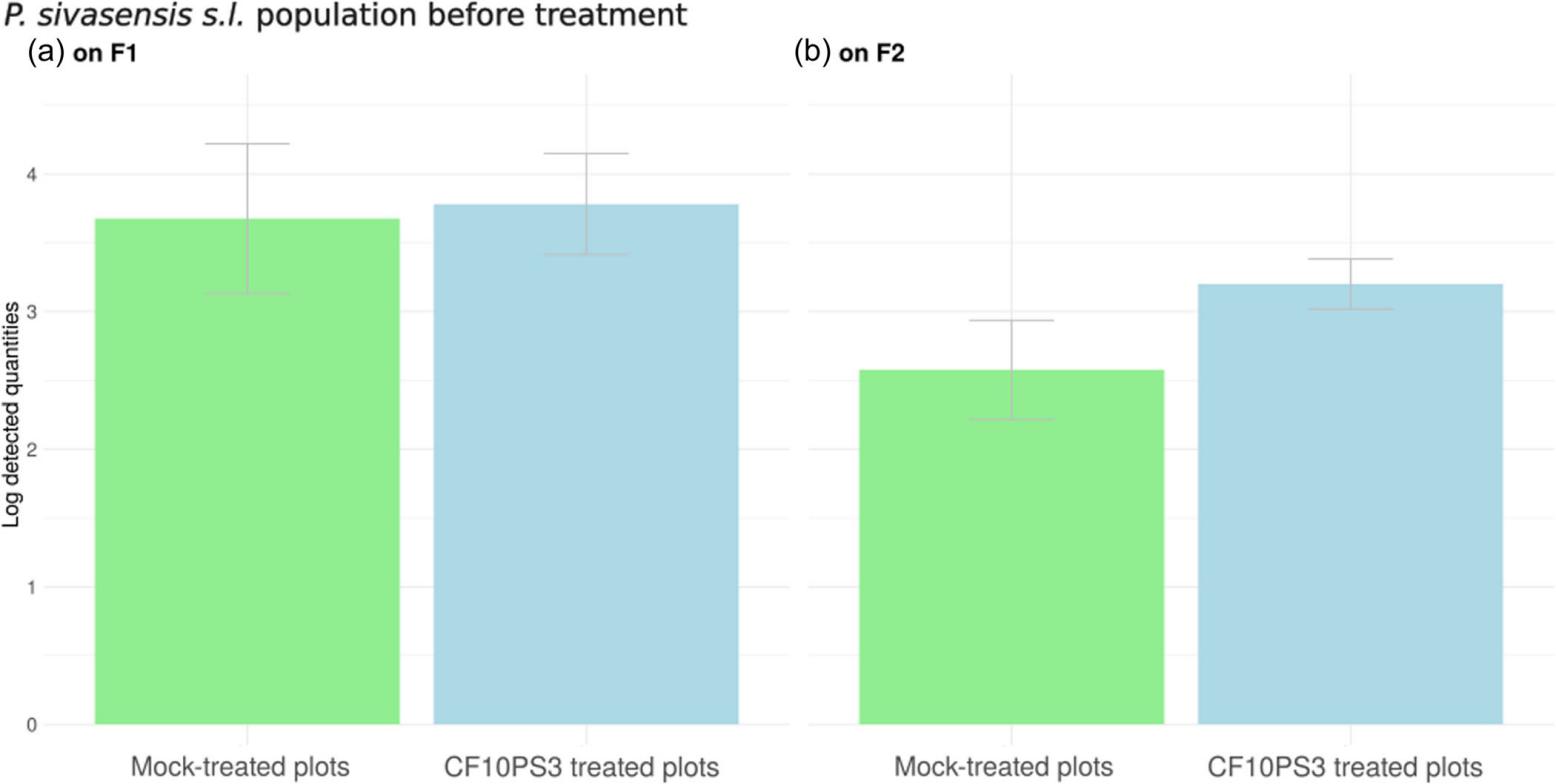
Natural population levels before treatments on F1 (a) and F2 (b). Data are mean qPCR copies per gram of leaf, corresponding to the natural population of *P. sivasensis* sensu lato from field plots (*n* = 4), either mock, treated with only buffer (green), or where CF10PS3 was applied (blue), along with their respective SD (bars).

**Figure 3:**
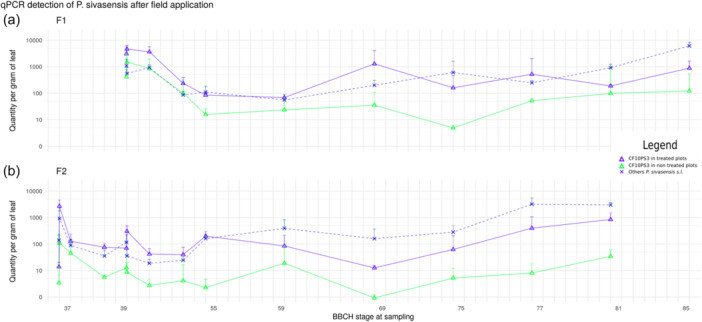
qPCR detection of *P. sivasensis* natural population and strain CF10PS3 on F1 (a) and F2 (b). Points are mean qPCR copies per gram of leaf from CF10PS3 (triangles) or natural population of *P. sivasensis sensu lato*, encompassing closely related species (crosses) represented for plots (*n* = 4) mock treated with only buffer (green), or where CF10PS3 was applied (blue), along with their respective SD (bars). For the natural population analysis, data from bacteria‐treated and mock‐treated plots showed no significant statistical difference and were therefore combined for this representation.

In this field experiment, natural populations of *P. sivasensis s.l.* were comparable across all plots and on F1 and F2, with no statistical difference (Figure 2). The strain of interest was already present on the leaves before the initial treatment targeting F2 leaves on May 11 (foliar stage BBCH37). Indeed, a slight level of detection (4 copies per gram of leaf) of CF10PS3 was observed in plots where the bacterium had not been applied yet (Fig. 3b, green triangle). This could be expected, considering that the strain was isolated from a flag leaf, indicating its natural presence and adaptation to the foliar cereal leaf environment. Higher levels of detection, with about 10 copies per gram (Figure 3b, blue triangles), were observed in plots where a previous treatment targeting F3 had been applied at BBCH32, 15 days before the first sampling of second leaves. At that time, the developing F2 leaves were probably impacted, with bacteria in the spray application likely landing on emerging F2. With very few exceptions, CF10PS3 can be detected in around 10 copies in plots where it was not applied.

#### 3.3.2 Second Leaf Bacterial Population Dynamic

**Figure A1:**
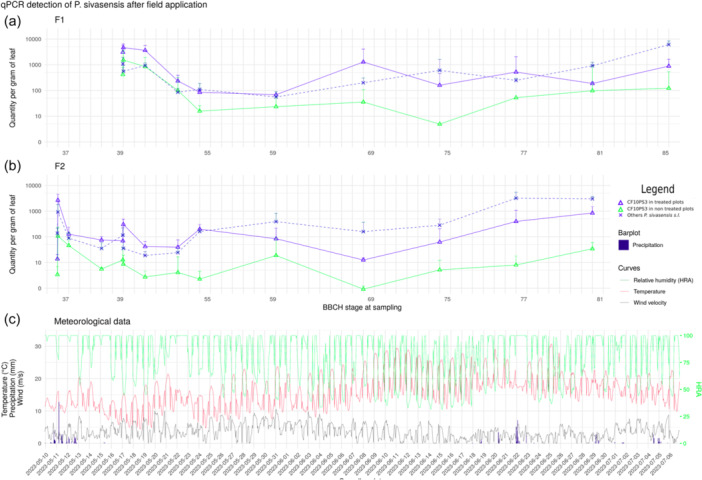
qPCR detection of *P. sivasensis* in the field, with meteorological data. Points are mean qPCR copies per gram of leaf from CF10PS3 (triangles) or natural population of *P. sivasensis sensu lato*, encompassing closely related species (crosses) represented for plots (*n* = 4) mock treated with only buffer (green), or where CF10PS3 was applied (blue), along with their respective SD (bars). For the natural population analysis, data from bacteria‐treated and mock‐treated plots showed no significant statistical difference and were therefore combined for this representation.

Following the first treatment targeting F2 on May 11 (BBCH37), a significant increase in CF10PS3 presence on treated leaves was noted, with a 100‐fold increase (Figure 3b, blue triangles). A slight 10‐fold increase was also observed in untreated plots (green triangles), likely due to the wind drift during windy conditions at that moment of the day (Appendix Figure A1).

Subsequent sampling conducted the day after treatment revealed a substantial decrease in bacterial detection across all plots. A 13‐mm rain event, occurring 3 h after treatment (Appendix Figure A1), likely contributed to this decrease by washing off both applied and natural CF10PS3 populations. Additional rain episodes in the following days may further explain the continuous decrease in all plots, indicating potential wash‐off and dilution of bacterial populations. Bacterial levels were maintained until a new application on May 17 (BBCH39), aiming to protect F1. This treatment resulted in a 10‐fold increase of CF10PS3 in plots treated with bacteria (Figure 3b, blue triangle) since underlying F2 leaves were also impacted by the treatment targeting F1 leaves. Mock‐treated plots were unaffected, and surprisingly, natural populations decreased by 10 times (blue crosses). In the following two days (May 18 and 19), CF10PS3 populations dropped by a 10‐fold factor, while natural populations remained stable. Windy conditions with lower humidity might have increased desiccation for the bacteria (Appendix Figure A1).

Over the next 3 days (until May 22), wind persisted, but with milder temperatures and higher humidity, potentially allowing bacteria to maintain their levels. Throughout the subsequent 5 weeks, all populations followed similar increasing trends, with natural populations consistently and statistically close in number. At the end of the sampling campaign, detected amounts of CF10PS3 in plots treated with bacteria were 20 times higher than in mock‐treated plots, ranging from 50 copies per gram to nearly 1000.

#### 3.3.3 First Leaf Bacterial Population Dynamics

On F1 leaves (Figure 3a), initial amounts of CF10PS3 were notably high, around 1000 copies per gram on May 17 (BBCH39). Unsurprisingly, there was a 10‐fold difference between treated and untreated plots. Natural populations remained significantly consistent across all plots after bacterial application. A significant drop in all bacterial populations was observed in all plots until May 22. In the following 5 weeks, all curves followed a similar pattern, also observed on F2 leaves. At the end of the sampling campaign, natural *P. sivasensis s.l.* populations reached approximately 5000 copies, whereas plots where CF10PS3 was applied or the untreated plots showed 1000 and 100 copies per gram, respectively. Copies of CF10PS3 were consistently higher throughout the season when applied in the field compared with mock‐treated plots.

#### 3.3.4 Seasonal Dynamics

The natural population levels of *P. sivasensis* and closely related species fluctuated between 500 and 5000 copies per gram during the sampling campaign, conducted from the foliar stage BBCH37 to the senescence of F1 and F2 leaves (BBCH85 and 81, respectively). When CF10PS3 was applied, there was a notable increase in copies throughout the season, with fluctuations ranging between 10 (F2) or 50 (F1) and 5000. Ultimately, the number of CF10PS3 copies was significantly higher in plots where the bacteria were applied compared to mock‐treated plots. Foliar application can influence the bacterial population of a single species throughout the entire season.

## 4 Discussion

*P. sivasensis* CF10PS3, a common bacterial species in small grain cereals, was isolated from a spelt field in Walhain, Belgium, under organic management. It produces biocontrol‐related metabolites, and can antagonize wheat pathogens, particularly *Z. tritici*. CF10PS3 is distinct from other *P. sivasensis* found in wheat fields, as it has an SNP in the 16S‐ITS‐23S operon that allowed us to design a qPCR experiment to specifically detect it.

The durability of a plant protection control method is crucial for its long‐term efficacy, defined as its persistence in space and time (Bardin et al. 2015). However, various biocontrol agents (BCAs) have shown inconsistent efficacy under commercial field conditions, proving less effective or even ineffective compared to controlled conditions (Bonaterra et al. 2022). Traditionally, the persistence of introduced bacteria was assessed using serial dilution and plating on selective agar media, which did not allow discrimination between introduced strains and related natural populations (Elad and Kirshner 1993; Paul et al. 2017; Pujol et al. 2006). In this study, we developed qPCR probes to distinguish the introduced *P. sivasensis* strain CF10PS3 from naturally present *P. sivasensis s.l.* in the wheat phyllosphere. This tool was useful to investigate the persistence of the introduced strain over 7 weeks post‐application. The results also indicated the absence of a noticeable effect of the CF10PS3 introduction on wild *P. sivasensis* naturally present on leaves.

Similar experiments had already been conducted on strawberry, following the colonization of Methylobacterium extorquens and its impact on fruit flavor (Verginer et al. 2010), and on potato and black pepper to examine the endophytic colonization of *P. putida* strain P9 (Andreote et al. 2009) and BP25 (Agisha et al. 2017), respectively. Other works have developed strain‐specific qPCR for soil and root‐associated microorganisms (Mendis et al. 2018).

Our field study suggests that environmental factors, particularly rainfall and humidity, may play a significant role in shaping the dynamics of bacterial populations, including *P. sivasensis* strain CF10PS3. While the strain has demonstrated an ability to persist in the wheat phyllosphere, fluctuations observed posttreatment point to the possibility that environmental conditions could impact both introduced and naturally occurring bacterial populations. This raises the importance of carefully considering the timing of bacterial treatments to enhance colonization and efficacy. The decreases in CF10PS3 populations observed after treatment could plausibly be associated with weather events, such as rainfall, which may have contributed to bacterial wash‐off. Such patterns hint at the sensitivity of bacterial persistence to external factors, although further studies would be required to definitively link these changes to specific meteorological conditions. The potential for population recovery following these environmental challenges suggests a certain resilience in CF10PS3, a characteristic that may be beneficial for its continued development as a biocontrol agent.

Additionally, our use of strain‐specific probes allowed precise tracking of the introduced strain, distinguishing it from other *P. sivasensis* strains naturally present in the field. This level of specificity is vital for understanding the persistence and behavior of the strain in a complex field environment. Despite the variability in population levels, plots treated with CF10PS3 generally maintained higher bacterial counts compared to control plots. This observation indicates that targeted bacterial applications can positively influence foliar bacterial populations, even under fluctuating environmental conditions. Investigating methods to enhance the strain's environmental stability, perhaps through formulation or application timing adjustments, could further improve its potential in sustainable crop management.

The primary drawback of qPCR detection lies in the presence of non‐degraded DNA in samples, with the rate of DNA degradation post‐cell death being highly dependent on environmental conditions (Nielsen et al. 2007). While research has explored DNA persistence in soil, varying results have been reported, ranging from rapid degradation in some studies (Nielsen, Smalla, and Van Elsas 2000) to prolonged persistence in others (England, Lee, and Trevors 1997). Assessing DNA persistence in the phyllosphere requires further investigation. Nevertheless, our field trial results suggest that long‐term DNA stability is unlikely in the phyllosphere. Two and 5 days after F1 field inoculation, all treated plots exhibited a 1‐ and 2‐log decrease, respectively, in CF10PS3 population levels, indicating rapid population decline. In this case, the rain wash‐off hypothesis can be excluded since no precipitation was recorded during these days.

Monitoring the presence and persistence of BCAs allows for the evaluation of their effectiveness in controlling plant pathogens under field conditions and their impact on native epiphytic communities. Understanding how BCAs react to environmental conditions enables researchers to optimize application strategies, including timing, reapplication, dosages, and formulations for more effective pest control. Assessing the interaction between different co‐formulants and BCAs will help determine their influence on efficacy, stability, and persistence. This information is crucial for optimizing formulation combinations and identifying synergistic effects that enhance overall performance. Understanding the impact of co‐formulants will contribute to the development of commercially viable biocontrol products.

The inconsistent efficacy observed in field settings, despite promising results in controlled conditions, underscores the need for a comprehensive understanding of BCA persistence. Traditionally, assessing BCA persistence relied on techniques unsuitable for discriminating between introduced strains and natural populations. Our innovative use of qPCR probes allowed us to overcome this limitation, specifically distinguishing the introduced *P. sivasensis* strain CF10PS3 from its naturally occurring counterpart in the wheat phyllosphere. This technological advancement enabled a detailed investigation of the persistence of the introduced BCA candidate for 7 weeks. Comparing our detected levels with those reported in the literature is challenging due to the limited available data. However, Pujol et al. (2006) also observed a decline in populations of *Pseudomonas* spp. after application, with stabilization at approximately 5000 CFU per gram, which aligns with our findings of population stabilization at around 1000 copies per gram. Similarly, Paul et al. (2017) reported a reduction in *Bacillus thuringiensis* populations from 10^12^ to 10^4^ UFC within 6 days post‐application, indicating that a decline postinoculation is typical for bacterial biocontrol agents, yet sufficient numbers can remain to confer beneficial effects.

As for the comparison between F1 and F2 inoculation, we observed that the bacterial population level on F1 leaves before application was already higher than on F2 leaves. This is in line with the origin of the strain, which was isolated from a flag leaf (F1), suggesting its natural adaptation to this part of the plant. On F2 leaves, after the initial introduction of the bacteria, the strain quickly established itself, occupying approximately 50% of the *P. sivasensis* population, as both signals were at the same levels. At growth stage 59, the strain still accounted for around 10% of the population, and this proportion was maintained until the last sampling. On the flag leaves (F1), the introduced strain was the dominant population for the first 3 weeks post‐application, after which it stabilized at around 10% of the total population. These levels are promising, as they suggest that the strain is well‐adapted to the leaf environment and can persist at levels that are hopefully sufficient to control diseases.

However, the bacterial load required for effective disease control is a crucial factor that must be thoroughly investigated. Future studies should focus on determining the optimal bacterial concentrations needed for disease suppression in similar environments. This could involve testing varying application concentrations to better understand the relationship between bacterial load and disease control efficacy. Such studies are essential to substantiate any strain's potential as a biocontrol agent and to optimize its practical application in the field. Given the progression dynamics of STB, which spreads from the lower leaves to the upper leaves via rain splash, the introduced strain must be able to colonize all foliar levels. This is particularly important for protecting the flag leaves (F1), which contribute the most to wheat yield, as well as the second leaves (F2) to a lesser extent. Ensuring effective colonization of both leaves could provide critical protection against STB, maintaining healthy foliage and preserving crop yield potential.

The phyllosphere's dynamic nature, influenced by factors such as moisture, temperature fluctuations, UV radiation, and nutrient availability, presents a challenging environment for microorganisms. Despite these hurdles, our field experiment, targeting F1 and F2 leaves with a strain applied in a buffer, demonstrated a significant increase in *P. sivasensis* presence throughout the season. Transitory decreases, attributed to rain events and environmental factors, underscored the dynamic nature of bacterial populations. Despite fluctuations, the introduced bacteria exhibited persistence, hinting at its potential as a protective agent. Here, it was demonstrated that it can also directly colonize leaves after foliar application, suggesting a potential for biocontrol application.

Our research highlights the importance of tracking BCAs on leaves. This capability will help optimize application strategies and allow for the study of co‐formulants, improving biocontrol methods. Overall, this research increases our understanding of BCA behavior in complex field environments, leading to more effective and adaptable plant protection strategies.

## 5 Conclusion

Our study underscores the potential of the *Pseudomonas sivasensis* CF10PS3 strain as a biocontrol agent in wheat fields, particularly against the pathogen *Z. tritici*. The development of qPCR probes enabled precise monitoring of CF10PS3, distinguishing it from naturally occurring *P. sivasensis*, and provided insights into its persistence and dynamics in the field. The results demonstrated that despite environmental challenges such as rainfall and temperature fluctuations, the CF10PS3 strain maintained a higher presence in treated plots compared to controls, suggesting its potential for effective foliar application. The ability of CF10PS3 to persist and adapt to the phyllosphere environment, both as a seed coating and a foliar application, highlights its versatility as a biocontrol agent. This study also emphasizes the need for optimizing application strategies, including timing and formulations, to enhance the efficacy and stability of biocontrol agents under variable field conditions. Understanding the interactions between biocontrol agents and environmental factors, as well as co‐formulants, is crucial for developing commercially viable and effective biocontrol products. Overall, our research contributes to a deeper understanding of biocontrol agent behavior in field environments, advocating for the implementation of targeted bacterial applications in plant protection. This knowledge will aid in improving biocontrol methods, ultimately leading to more sustainable and resilient agricultural practices.

## Author Contributions

**Mathieu Delitte:** conceptualization (lead), formal analysis (lead), writing–original draft (lead). **Benjamin Dubois:** conceptualization (supporting), formal analysis (equal), writing–original draft (supporting). **Jacques Mahillon:** conceptualization (supporting), writing–review and editing (supporting), funding acquisition (equal). **Frédéric Debode:** conceptualization (supporting), writing–review and editing (supporting), funding acquisition (equal). **Claude Bragard:** conceptualization (supporting), writing–review and editing (lead), funding acquisition (equal).

## Ethics Statement

The authors have nothing to report.

## Conflicts of Interest

*P. sivasensis* strain CF10PS3 is under the patenting process (BE2024/5419) and might be used as a BCA by the UCLouvain spin‐off company BIOCSOL.

## Data Availability

The data set generated and analyzed during the current study is openly available in the Zenodo repository: https://doi.org/10.5281/zenodo.12633665.

## 1

**Table A1:** Number of sequences used in the alignment for probes and primers design and off‐target detection.

Species	Nb of sequences	Nb of strains	Off‐target strains
*P. arvensis* sp. nov.	24	4	/
*P. chlororaphis*	36	7	/
*P. cyclaminis*	6	1	/
*P. fluorescens*	92	22	2020WEIHUA
			ATCC13525
			NCTC10038
*P. germanica*	4	1	/
*P. granadensis*	4	1	/
*P. lactis*	11	6	/
*P. lurida*	15	5	/
*P. marginalis*	21	5	/
*P. orientalis*	13	7	/
*P. poae*	35	11	DSM14936
			LMG21465
			PA1‐8B
			PA2‐1F
			RE1114
			SWRI2
			WS5103
*P. salmasensis*	6	1	/
*P. simiae*	12	4	/
*P. sivasensis*	36	8	/
*P. tensinigenes*	4	1	/
Total	319	84	10
